# Retrospective exposure assessment to airborne asbestos among power industry workers

**DOI:** 10.1186/1745-6673-5-15

**Published:** 2010-06-25

**Authors:** Michael K Felten, Lars Knoll, Christian Eisenhawer, Diana Ackermann, Khaled Khatab, Johannes Hüdepohl, Wolfgang Zschiesche, Thomas Kraus

**Affiliations:** 1 Institute of Occupational and Social Medicine, Medical Faculty, RWTH Aachen University, Aachen, Germany; 2 Institute of Medical Statistics, Medical Faculty, RWTH Aachen University, Aachen, Germany; 3 Institution for Statutory Accident Insurance and Prevention in the Energy, Textile, Electrical, and Media Industry (BGETEM), Cologne, Germany

## Abstract

**Background:**

A method of individually assessing former exposure to asbestos fibres is a precondition of risk-differentiated health surveillance. The main aims of our study were to assess former levels of airborne asbestos exposure in the power industry in Germany and to propose a basic strategy for health surveillance and the early detection of asbestos related diseases.

**Methods:**

Between March 2002 and the end of 2006, we conducted a retrospective questionnaire based survey of occupational tasks and exposures with airborne asbestos fibres in a cohort of 8632 formerly asbestos exposed power industry workers. The data on exposure and occupation were entered into a specially designed computer programme, based on ambient monitoring of airborne asbestos fibre concentrations. The cumulative asbestos exposure was expressed as the product of the eight-hour time weighted average and the total duration of exposure in fibre years (fibres/cubic centimetre-years).

**Results:**

Data of 7775 (90% of the total) participants working in installations for power generation, power distribution or gas supply could be evaluated. The power generation group (n = 5284) had a mean age of 56 years, were exposed for 20 years and had an average cumulative asbestos exposure of 42 fibre years. The occupational group of "metalworkers" (n = 1600) had the highest mean value of 79 fibre years. The corresponding results for the power distribution group (n = 2491) were a mean age of 45 years, a mean exposure duration of 12 years and an average cumulative asbestos exposure of only 2.5 fibre years. The gas supply workers (n = 512) had a mean age of 54 years and a mean duration of exposure of 15 years.

**Conclusions:**

While the surveyed cohort as a whole was heavily exposed to asbestos dust, the power distribution group had a mean cumulative exposure of only 6% of that found in the power generation group. Based on the presented data, risk-differentiated disease surveillance focusing on metalworkers and electricians from the power generating industry seems justified. That combined with a sensitive examination technique would allow detecting asbestos related diseases early and efficiently.

## Background

Asbestos dust is a serious health hazard leading to diseases such as asbestosis, lung cancer and mesothelioma of the pleura. The first cases in an exposed population may appear as soon as five years after the beginning of exposure [1,2]. The silent period of several years or decades, known as the latency period of asbestos disease, creates a specific problem when planning health surveillance programmes based on risk of disease. As most would agree that disease risk is somehow related to the inhaled amount of asbestos dust, a method of individually assessing former exposure is a precondition of risk-differentiated surveillance several years later [3].

The use of asbestos in Germany developed after the end of the Second World War similar to other industrialised countries [4-6]. After a steady rise from around 1950, consumption in Western Germany (Federal Republic of Germany, FRG) peaked in the year 1977, in Eastern Germany (German Democratic Republic, GDR) around 1980. Although some regulations for the reduction of asbestos dust had already been applied from the mid 1930s, employees usually handled asbestos materials without any effective personal protection. In 1979, the spraying of asbestos pulp, one of the most dangerous methods of processing asbestos, was banned in the FRG. A series of additional regulations limiting the use of asbestos led to the total ban of the import and processing of asbestos and all types of asbestos containing materials (ACM) from the beginning of 1993.

For most work places where high temperatures or the need for heat protection demanded the use of insulation materials, a significant asbestos risk can be assumed. Especially employees in electricity generating power plants and installations for power distribution were consistently exposed to airborne asbestos fibres [7-11]. Formerly asbestos exposed workers in the power industry must therefore be seen as a high-risk group for asbestos related disease [12-14]. However, little is known on the actual cumulative exposure related to specific occupations in the power industry. Reported assessments of workplace exposure levels in power plants and similar occupations are scarce and difficult to compare [15-19]. Some of the reported results may lead to a significant underrating of the exposure levels formerly prevalent in the power industry. Ambient monitoring in power plants for instance not including high exposure periods, such as turbine revisions, or outside the working areas where ACMs were handled are of little value for identifying high risk occupational groups. Specific risks of asbestos exposure in installations for power distribution are virtually unknown in the published literature [14]. When considering risk-differentiated health interventions among asbestos exposed workers, particularly the impact and costs of such interventions, a good understanding of the range of individual disease risks is essential. In spite of known shortcomings and likely biases [20-24], a serious attempt for a job specific, retrospective occupational exposure assessment among power industry workers is therefore urgently needed.

## Methods

We conducted a questionnaire-based survey of 8632 formerly or still active employees of a major provider of electrical power in Germany. In this survey, we evaluated data on self-reported exposures with asbestos fibres stratified by the participants' job titles and main occupational tasks. The majority of participants were employed in one of eight electricity-generating power plants. Six of the power plants used conventional fuel, mainly lignite or coal, and the other two were nuclear power plants. Most of the remainder worked in various installations for power distribution such as transformer stations, open mining plants for lignite, in a variety of outlaying workshops or they executed special functions, for example as fire fighters, security personnel or warehouse workers. The rest of the cohort was a small group of formerly asbestos exposed gas supply workers. They had been identified and enrolled with some delay, so that their detailed exposure data and job tasks could not be included in this evaluation.

Enrolment for the survey was originally started as an internal programme organised by the company medical officers and beginning in the late 1990s. All persons, who could be contacted and replied by submitting a signed statement that they had been exposed to asbestos fibres, were enrolled for the survey. Enrolment continued until the end of the year 2006, when the survey was closed for new admissions. From March 2002, we invited the participants for routine screening examinations in accordance with regulations by the Institution for Statutory Accident Insurance and Prevention in the Energy, Textile, Electrical, and Media Industry (BGETEM). We will publish the results of these health examinations in a companion paper at a later stage.

The presented exposure data are based on job titles, length of exposure and specific occupational tasks. That information was collected by means of a specially designed self-administered questionnaire, which had been mailed to the participants before examination. The questionnaire items concentrated on the type of occupation and periods with likely exposure to airborne asbestos fibres, including previous periods of exposure before participants had joined the company or while still in training. Periods of exposure indicated in the questionnaires for the time after 1992 were not included in the evaluation. While we cannot rule out unprotected exposure to asbestos dust for short periods after 1992, fibre concentrations in that time would not have reached levels comparable to those in the years before asbestos was totally banned. In addition to typical tasks, such as removal of asbestos insulation, spraying of asbestos pulp and removal or installation of gaskets and packings, explicitly mentioned in the questionnaire, there was also room for additional remarks. Other more general items referred to the periods of participation in the manual removal and replacement of asbestos insulation. Participants, who belonged to parts of the company not involved in running or servicing power plants, received a modified version of the questionnaire with no direct reference to job tasks typical for a power plant. The majority of these employees were electricians, who had worked in installations for power distribution and doing routine tasks such as tooling of asbestos cement sheets with a saw or drill, or handling of asbestos cords or boards. As a result we obtained two sets of questionnaires, one for the subgroup of power generation workers with information on asbestos exposure and occupational tasks mostly related to routine turbine revisions and a second set for the power distribution workers, who were not involved in turbine revisions but routinely handled ACMs in a variety of other job tasks.

The Central Registration Agency for Employees Exposed to Asbestos Dust (GVS) provided an additional list of all participants with allocated job titles, some information on the use of protective measures at the asbestos exposed workplaces and the use of different fibre types. The allocation of job titles as indicated in the GVS list and providing the other information was part of the registration process by the responsible medical officers of the company. Relevant job titles were those applicable for the most recent jobs with exposure to asbestos dust. At the time of enrolment, most former employees were already retired or in the status of pre-retirement. Few participants had moved to other companies or they were self-employed.

The original database of the cohort included 338 different job titles describing specific functions like welder, mechanic or pipe fitter. Job titles provided by the GVS were entered into that database with first priority. In cases of discrepancy the job titles from the GVS list were used for evaluation. Only in participants without information from the GVS, job titles from the self-administered questionnaires were used. The total number could be reduced to 91 unique job titles after eliminating redundancies due to spelling variations, abbreviations, multiple code numbers or the parallel use of outdated titles [25]. The 91 remaining job titles were allocated to six basic occupational groups with similar functions and similar exposures to asbestos dust, which defined the similar exposure groups (SEGs) for the purpose of this evaluation. The final result was reached in cooperation with a panel of occupational safety experts, who were accustomed to the use of historical or unusual job titles and tasks in the power industry.

The allocation to the SEGs was a stepwise process based on the assumption that craftsmen working close to the turbines had the highest risk of asbestos exposure and those not occupied as craftsmen the lowest. Consequently, all job titles with the main function of metal working, particularly the welders, insulators and mechanics, were allocated to one group ("metalworkers") with probably high exposure levels. From the remaining job titles, all those referring to a function in the field of electrical or electronic work were allocated to the common SEG of "electricians". This group did however not include job titles such as electronic engineers, because their typical function was clearly different from that of an industrial electrician as far as asbestos exposure was concerned. The third exposure group of "plant operators" included all job titles referring primarily to system controlling with no routine handling or tooling of ACMs, but constantly working inside the turbine halls next to the craftsmen handling asbestos and ACMs. Out of the rest of job titles, those referring to any of a wide variety of crafts and trades were grouped together under the SEG label "other craftsmen" with an increased, but probably inhomogeneous exposure risk. For the remaining non-craftsmen a decision was made whether they had mainly supervising or planning functions ("supervisors") with job titles like work planner, civil engineer or physicist, or they fell into the last group "other occupations" with unspecific job titles such as receptionist, security personnel or office staff.

Before descriptive analysis, we carefully checked all survey data for plausibility and correctness. Missing or implausible values were verified with the original documentation collected in the central archive of the survey in Aachen. The data analysis for this paper was generated using SAS software, Version 9.1 of the SAS System for Windows (Copyright © 2002-2003 SAS Institute Inc). The data set obtained from the self-administered questionnaire was entered into a computer programme provided by the BGETEM, which allowed calculating the cumulative individual asbestos exposure in a standardized way. The software had been developed by a panel of occupational safety experts and was based on ambient monitoring data of airborne asbestos fibre concentrations at defined workplaces, stratified by typical occupational tasks and time-periods. The basic reference data have been published by the BGETEM in the format of a technical report [26]. The ambient monitoring data used in the report covered a period of four decades from the beginning of the 1950s until 1990 [4]. In the 1970s, the original technique of konimetrical measurements was gradually replaced with membrane filter techniques applying a defined airflow. Both methods were not specific for asbestos fibres. That deficit was overcome by combining membranous filtering systems with the microscopical count of fibres. The software for calculating individual cumulative exposures was operated through an easy-to-use graphic user interface to be run on a common stand-alone personal computer. The cumulative asbestos exposure in that computer programme was expressed as the product of the eight-hour time weighted average fibre concentration and the total duration of exposure. (in fibres/cubic centimetre - years or "fibre years"). The cumulative dose of one standard fibre year was defined as an exposure during 1920 work hours through daily eight-hour shifts over 240 workdays and spread over 48 weeks with a standard airborne fibre concentration of one fibre per cubic centimetre or 1 × 10 ^6 ^fibres per cubic metre.

For developing the fibre year model used in the software, typical occupational tasks with asbestos exposure had first to be identified for various types of industry, such as power generation or power distribution. By linking defined tasks, individual work periods and job specific monitoring data as published in the "BK-Report", it was then possible as a second step to calculate individual fibre year values. When for example for the typical task of spraying asbestos pulp an eight-hour time average fibre concentration of 400 fibres per cubic centimetre was assumed, a worker doing nothing else would have accumulated a total exposure of 400 fibre years during one standard calendar year of 240 workdays. To consider shorter periods than a full year, the total annual value for spraying asbestos pulp was subdivided into 1.67 fibre year increments per day (400 fibre years divided by 240 workdays of a standard calendar year). A worker participating in regular turbine revisions over 10 years with spraying periods of 30 workdays every year would have accumulated 300 workdays of spraying pulp. As certain start-up and set-up times were unavoidable, the spraying with full exposure was never done throughout shifts. The total fibre year value of 501 in this example would therefore be reduced to 20% of the calculated value as a standard correction for that task, resulting in a value of 100 fibre years cumulative asbestos exposure. For other typical tasks defined in the model, such as replacing asbestos containing gaskets or maintaining electrical appliances, adjusted average fibre concentrations (2.5 fibres per cubic centimetre) and correction factors were used. When applied to job tasks outside regular turbine revisions, fibre year increments per minute were used [26].

## Results

We concluded the survey among the enrolled 8632 asbestos exposed power generation and distribution workers by the end of the year 2006 (Table 1). As the 28 female participants represented only 0.3% of the total number, they were not analysed separately. The main characteristics of the three groups indicate that the power generation workers, who represented approximately two thirds (65%) of all participants, were 10 years older (55 versus 45 years) than the second largest group of the 2498 (29%) power distribution workers and had a much longer mean period of asbestos exposure (20 versus 13 years). The 512 gas supply workers (6% of the total) had a similar mean age of 54 years, but their mean period of asbestos exposure was with 15 years closer to the power distribution group. We based the calculation of age for all participants on the fixed reference date of 1 September 2002, which ensured that the date of investigation had no influence on the comparison of groups. The date was roughly marking the transition between the predominant activities of enrolment and investigation, but had no other significance. The mean periods of employment ranged from 20 years in the power generation group to 25 years in the gas suppliers (Table 1). The described differences of the three groups with the use of different data sheets and the lack of information for the gas supply workers made it necessary to analyse the groups separately. The detailed analysis of job tasks and related asbestos exposures had to be confined to the main groups of power generation and distribution workers, from whom completed questionnaires could be obtained. Of the 8120 participants in the power generation and power distribution groups 6165 (76%) returned fully completed questionnaires with information on job titles, duration of exposure and specific occupational tasks. Another 1610 (20%) participants provided sufficient information only on exposure periods and job titles, resulting in a total number of 7775 (96%) with enough data for calculating the individual cumulative exposure (Tables 2 and 3). For the gas supply workers, that information was not available for analysis. As job titles used by the company medical officers for registration were the same for all participants, exposure assessments could still be compared based on occupational exposure groups.

**Table 1:** Demographic characteristics of the three subcohorts (total cohort n = 8632)

	Power generation	Power distribution	Gas supply
**N**^**a**^	5622 (65%)	2498 (29%)	512 (6%)
**Mean age**^**b**^	55 (12)	45 (12)	54 (11)
**Period of employment**^**c**^	20 (12)	22 (9)	25 (9)
**Asbestos exposure**^**c**^	20 (10)	13 (8)	15 (9)

^a ^Percent of total cohort in parantheses

^b ^Mean age at the fixed reference date 1 September 2002, standard deviation in parentheses

^c ^Mean period in years, standard deviation in parentheses

### Power generation workers

When stratifying age, periods of exposure, fibre years and typical job tasks by the defined SEGs, certain differences within the power generation group became obvious (Table 2). We based the comparison between SEGs mainly on the average individual exposure estimates and the percentages of participants who carried out specific job tasks. The two largest exposure groups were the metalworkers (n = 1600) and the plant operators (n = 1588), both together representing 60% of the 5284 power generation workers, who gave usable information on job titles and periods of exposure (94% of the total group of 5622). The mean age was 56 years, ranging from 52 years in the electricians and other craftsmen to 59 years in the group of other occupations. The young age of the group of other craftsmen was reflected in their low mean values for exposure time (18 years) and fibre years (21 years). The metalworkers had 79 fibre years, which was an extremely high value in comparison to all other SEGs. The eight typical job tasks were represented in all six SEGs, ranging from 1% of the electricians formerly doing some spraying of asbestos pulp to 75% of the metalworkers actively involved in turbine revisions. For the other seven job tasks except one, the percentages in the metalworkers were also the highest. Only maintenance work with heat resistant wires was less common for metalworkers than the other SEGs, particularly the electricians (67%). When comparing the results of the six SEGs with those of the total group, the metalworkers, the electricians, and the plant operators were above average in at least two percentage values. The metalworkers and the electricians also had the highest fibre year values. The supervisors and those in the group with "other occupations" had low fibre years and were less involved with asbestos exposed tasks, but their results were still comparable with those of the other groups (Table 2).

**Table 2:** Power generation workers: similar exposure groups, cumulative exposures and job tasks (n = 5284; 94% of the total group). Evaluable data on specific job tasks were available in 70% (n = 3696) of the 5284 workers with usable data

Exposure group	n (%)	**Age**^**g**^	**Exposed**^**h**^	Fibre-	**Revisions**^**k**^	**Removal**^**k**^	**Spraying**^**k**^	**Gaskets**^**l**^	**Packs**^**l**^	**Mats**^**l**^	**Gauges**^**l**^	**Wires**^**l**^
		(years)	(years)	**years**^**i**^	**(%)**^**m**^	**(%)**^**m**^	**(%)**^**m**^	**(%)**^**m**^	**(%)**^**m**^	**(%)**^**m**^	**(%)**^**m**^	**(%)**^**m**^
Metalworker^a^	1600 (30)	54 (12)	21 (11)	79 (195)	75	28	7	65	56	37	46	5
Electrician^b^	652 (13)	52 (12)	20 (11)	33 (132)	63	11	1	16	7	12	18	67
Plant operator^c^	1588 (30)	56 (12)	20 (10)	26 (77)	51	24	4	28	23	27	21	6
Other craftsman^d^	601 (11)	52 (11)	18 (10)	21 (70)	47	17	2	24	19	22	17	14
Supervisor^e^	210 (4)	57 (10)	19 (10)	27 (99)	43	12	2	14	12	13	11	9
Other occupation^f^	633 (12)	59 (11)	21 (10)	22 (74)	50	14	4	22	19	19	21	12

Total	5284 (100)	56 (12)	20 (11)	42 (134)	59	21	4	36	30	26	28	15

^a ^including welder, insulator, mechanic

^b ^including communications technician

^c ^including system controller, boiler operator

^d ^for example roofer, pipefitter, car mechanic, mason, gauge mechanic, turner, smelter, fire fighter, driver, painter, warehouseman, forklift operator

^e ^present in the contaminated area, but not executing manual work, for example civil engineer, work planner

^f ^for example security personnel, office staff, receptionist

^g ^age at fixed reference date 1 September 2002, standard deviation in parentheses

^h ^periods of asbestos exposure from self administered data sheets, standard deviation in parentheses

^i ^cumulative asbestos exposure calculated for a standard airborne concentration of 1 × 10^6 ^fibres per cubic metre, standard deviation in parentheses

^k ^actively involved in revision of steam turbines, particularly removal of asbestos lagging and spraying of asbestos pulp

^l ^tooling and handling of asbestos containing gaskets, packings and gauges, removal of asbestos mats, maintenance of heat resistant wires

^m ^percent of exposure group with evaluable data executing specific job task

### Power distribution workers

The corresponding results for the 2491 enrolled power distribution workers with usable information on job titles (99.7% of the total group of 2498) showed a mean age of 45 years, ranging from 44 years in the electricians to 56 years in the plant operators (Table 3). The electricians were also the biggest SEG (n = 1875) representing 75% of the total group. The lower mean age in comparison with that of the power generation group (56 years) was reflected in a correspondingly low mean exposure time of 12 years for the power distribution group, which was only exceeded by the metalworkers with 15 years. All SEGs in the power distribution group had much lower values for the fibre years in comparison to the power generation workers, with a mean value for the group of 2.5 fibre years. While the plant operators in the power distribution group had more than double that value (5.1 years), their SEG represented less than 1% of the total group and had therefore no marked effect on the overall mean. The eight defined job tasks were represented in all SEGs except one, ranging from 10% of the metalworkers formerly involved in the maintenance of night storage heaters to 77% of the electricians, who had used a drill on asbestos cement sheets. None of the plant operators had ever been involved in the maintenance of night storage heaters. When comparing the results of the six SEGs with the overall values for the whole group, all defined job tasks were more common in the electricians than in the other SEGs. The percentage of the job task "handling of cords" (52%) was much higher in metalworkers than the average of the group of power generation workers (24%). The plant operators had a higher than average percentage value for the handling of cords. Similar to the results in the power generation group, only the electricians and the metalworkers were above average in at least two percentage values, which is also reflected in high fibre year values for these two SEGs. Other than the metalworkers in the power generation group, the electricians reached the highest percentage values. However, their overall exposure in fibre years was just about 7% of that found as an average in the power generation group and less than 9% of the electricians of that group. When comparing the mean fibre year values of both groups (Tables 2 and 3), the power distribution workers had a cumulative asbestos exposure of approximately 6% of that found in the power generation group.

**Table 3:** Power distribution workers: similar exposure groups, cumulative exposures and job tasks (n = 2491; 99.7% of the total group). Evaluable data on specific job tasks were available in 99% (n = 2469) of the 2491 workers with usable data

Exposure group	n (%)	**Age**^**g**^	**Exposed**^**h**^	Fibre-	**Drilling**^**k**^	**Sawing**^**k**^	**Grinding**^**k**^	**Other**^**k**^	**Cords**^**l**^	**Boards**^**l**^	**Heater**^**l**^
		(years)	(years)	**years**^**i**^	**(%)**^**m**^	**(%)**^**m**^	**(%)**^**m**^	**(%)**^**m**^	**(%)**^**m**^	**(%)**^**m**^	**(%)**^**m**^
Metalworker^a^	50 (2)	53 (12)	15 (9)	2.8 (6)	52	52	48	66	52	30	10
Electrician^b^	1875 (75)	44 (13)	12 (8)	2.8 (23)	77	72	58	72	25	27	38
Plant operator^c^	13 (< 1)	56 (11)	12 (8)	5.1 (9)	23	31	15	38	31	23	-
Other craftsman^d^	250 (10)	46 (11)	12 (8)	1.3 (3)	48	46	34	57	19	20	21
Supervisor^e^	160 (6)	46 (10)	11 (8)	1.9 (13)	48	46	31	58	24	22	34
Other occupation^f^	143 (6)	48 (12)	12 (9)	1.0 (2)	48	43	33	49	12	19	28

Total	2491 (100)	45 (12)	12 (8)	2.5 (20)	70	66	51	68	24	25	35

^a ^including welder, insulator, mechanic

^b ^including communications technician

^c ^including system controller, boiler operator

^d ^for example roofer, pipefitter, car mechanic, mason, gauge mechanic, turner, smelter, fire fighter, driver, painter, warehouseman, forklift operator

^e ^present in the contaminated area, but not executing manual work, for example civil engineer, work planner

^f ^for example gardener, office staff, laboratory assistant, doorman, occupation unknown (n = 7)

^g ^age at fixed reference date 1 September 2002, standard deviation in parentheses

^h ^periods of asbestos exposure from self administered data sheets, standard deviation in parentheses

^i ^cumulative asbestos exposure calculated for a standard airborne concentration of 1 × 10^6 ^fibres per cubic metre, standard deviation in parentheses

^k ^tooling of asbestos cement sheets with a drill, saw, angle grinder or some other instrument

^l ^handling of asbestos cords or boards, maintenance of night storage heaters

^m ^percent of exposure group with evaluable data executing specific job task

### Types of asbestos fibres

Regarding the use of different types of asbestos fibres, we evaluated the information the company medical officers had recorded as part of the registration process for 5637 (65%) out of the 8632 participants. A large proportion of 40% (3479 participants) was exposed to both chrysotile and crocidolite fibres. Another 18% (1545 participants) were exposed to chrysotile only and the remaining 7% (613 participants) had only contact with crocidolite.

## Discussion

In our evaluation, we have considered a cohort of 8632 formerly asbestos exposed workers employed in industrial plants for power generation, power distribution and gas supply, which is one of the largest cohorts ever enrolled in the asbestos industry. The participants, from whom information on job titles, periods of exposure and typical job tasks could be obtained, represented a group of asbestos exposed workers whose names were registered and had a valid postal address. Considering the extended and diverse periods between the beginning of the participants' individual asbestos exposures and our survey, generalized conclusions on the characteristics of the original group of asbestos exposed workers and the work standards should be drawn with caution. These limitations did however not interfere with the main purpose of our survey, to relate historical cumulative asbestos exposure as assessed for different occupational groups to living workers' additional risk of developing asbestos related disease. We also considered the element of self-assessment in our approach more as an advantage than a weakness [22,27-29]. It is however clear that final assessment will depend on the outcome of a validation process by correlating exposure indicators, such as fibre years, with disease rates.

For the analysis presented here, the study group had to be divided into two main subgroups, the power generation and the power distribution workers. As the most important difference between these two groups was the involvement in routine revisions of steam turbines, we can assume that the third group of gas supply workers would be similar to the power distribution group. Because of the different categories of job tasks, we could not directly compare the two subgroups and valid conclusions applicable to both groups on the level of occupational tasks would be difficult. As the system of allocating job titles was uniformly applied to all participants, it was still possible to define the same SEGs for both groups. This together with the standardized method of calculating fibre years resulted in a unique database linking occupational data, age and standardized estimates of the cumulative exposure to asbestos fibres in 7775 power industry workers. The high proportion of 96% of the combined power generation and distribution groups with usable data on exposure history and job titles indicated a recruitment process effective in finding those who were most concerned about their health.

We allocated job titles to SEGs without considering changes of jobs or functions over the years and in what part of the plant or workshop the workers were stationed most of the time. The large standard deviations for both exposure time and fibre years, and the fact that the results for the group of supervisors were comparable with those of the craftsmen, are explained by this to some extent. Another explanation for the often larger variation of cumulative exposure (in fibre years) within SEGs than among SEGs was the variation of task profiles within the defined SEGs. As the fibre year calculations were based on task specific monitoring data, vastly different combinations of tasks within a specific SEG would result in a large variation of fibre year values, as shown in the results. The possible conclusion to base the definition of SEGs better on task profiles rather than on industries and job titles is in our view however misleading. SEGs based on task profiles may theoretically distinguish the levels of asbestos exposure with better precision, but as workers are usually grouped by industries and jobs rather than specific combinations of occupational tasks that approach seems impractical. Our definition of SEGs should in any case not be seen as an instrument of predicting individual cumulative exposures to asbestos, but rather as a general risk indicator for groups of workers with a common professional background. The percentages of participants falling into the defined SEGs reflect the characteristic combination of occupational tasks in the two main groups. The power generation group was mainly represented by the metalworkers and plant operators making up for 60% of the total group and showing a high cumulative asbestos exposure as indicated in long mean exposure times (21 and 20 years) and high fibre year values (79 and 26 years). The power distribution group was dominated by the electricians representing 75% of the group with a smaller cumulative asbestos exposure, as indicated in a short mean exposure time (12 years) and a fibre year value of only 2.5.

The validity of fibre year calculations depend on the quality of individual exposure data and that of the database linking defined workplaces and job tasks with fibre concentrations. In an individual case, calculating a numerical fibre year value as a measure of cumulative asbestos exposure may not necessarily imply a better precision than roughly estimating a certain level of exposure. The fibre year model in combination with a standard questionnaire and an easy-to-handle software tool seems however far superior when organising disease surveillance programmes or epidemiological surveys. Even a certain degree of imprecision on the individual level would not preclude conclusions for the whole group, which are more useful as a planning tool.

The data on exposure history and specific occupational tasks had been collected by means of self-administered data sheets with return rates of usable data ranging from 59% in the group of "other occupations" of the power generation group to 97% and more in the SEGs of the power distribution group. While we have no reason to believe that the obtained overall return rate of 70% in the power generation group resulted in a selection bias or we were dealing with an undisclosed recall bias, we cannot rule out the possibility that the validity of the calculated fibre years were weakened by these effects. The average fibre concentrations the BGETEM used for the calculations were based on ambient monitoring data of well-defined workplaces with known asbestos exposure in power generation plants and installations for power distribution, stratified by time-periods and occupations. The calculations were carried out without corrections for the use of personal or technical protective measures, such as filter masks or technical ventilation. As 82% of the examined power generation workers were registered with the information that no protective measures had been applied at their workplaces, the use of uncorrected monitoring data seemed justified. The one typical and widely used protective measure mentioned in the occupational histories from the 1960ties and 1970ties was a wet sponge or cloth pressed to mouth and nose. Our data on the fibre types used seem to indicate that in the power industry the proportion of workers exposed to crocidolite (approximately half the cohort) was probably higher in comparison to most other industries. An internal technical report used by the BGETEM ("Coenen, Schenk: BIA-Report 3/84") indicated that around 96% of dust samples from a wide variety of workplaces contained only chrysotile fibres. The samples for that evaluation had been collected in the years 1981 and 1982. We have no evidence that the 35% participants without records on exposure to specific fibre types were in contact with other combinations of asbestos.

For the purpose of our survey, it was most important to use results from standardized calculations, based on historical monitoring data and blinded for any individually biased assessment. That way we could obtain an objective view on both the mean cumulative asbestos exposure among power plant workers and characteristic differences among defined exposure groups.

## Conclusions

In the period between the early 1950s to the end of the year 1992, workers in the power generation and power distribution industry in Germany were exposed to asbestos. From the wide range of workplaces and the variety of occupations concerned, we concluded that a vast number of employees were affected. When comparing power generation and power distribution workers it became obvious that the power generation workers were more exposed. Their subgroup of metalworkers reached the highest fibre year values of all SEGs defined for the two subcohorts. The power distribution workers had a mean cumulative asbestos exposure of approximately 6% of that found in the power generation group. Based on the assumption that a higher cumulative asbestos exposure leads to an increased risk of asbestos associated disease such as asbestosis or lung cancer, we consider the occupational group of metalworkers in the power generation industry as a high-risk group. Comprehensive surveillance measures in power industry workers should be based on focussed epidemiological surveys taking into account age and occupation-specific disease risks. By applying that approach together with a sensitive examination technique, risk-differentiated, effective disease surveillance in different cohorts with typical risk patterns seems possible. Standardized fibre year calculations as routinely applied in this study are useful for epidemiological surveys aiming at an objective exposure assessment.

## Competing interests

The authors declare that they have no competing interests.

## Authors' contributions

MKF organized the cohort, managed the survey data and drafted the manuscript. LK, CE and MKF coordinated the examination of participants, extracted the relevant data for analysis and interpreted the results. JH, WZ and TK conceived the study, designed the building of the cohort and the framework of the survey, and discussed with an expert panel the definitions of historical job titles. DA and KK analyzed the study data and supported their interpretation. All authors read and approved the final manuscript.
